# Reduced egfr, elevated urine protein and low level of personal protective equipment compliance among artisanal small scale gold miners at Bibiani-Ghana: a cross-sectional study

**DOI:** 10.1186/s12889-017-4517-z

**Published:** 2017-06-27

**Authors:** Justice Afrifa, Samuel Essien-Baidoo, Richard K.D. Ephraim, Daniel Nkrumah, Daniel Osei Dankyira

**Affiliations:** Department of Medical Laboratory Technology University of Cape Coast, Cape Coast, Ghana

**Keywords:** Small scale mining, Mercury exposure, Renal function

## Abstract

**Background:**

Mercury is a toxic metal with its effects on human health ranging from acute to chronic in a very short time of exposure. Artisanal and small-scale gold mining (ASGM) is the main source of direct human exposure to mercury.

**Aim:**

To access the effect of mercury exposure on the renal function and level of personal protective equipment (PPE) compliance among small-scale gold miners in Bibiani District of the Western Region of Ghana

**Method:**

110 consenting male gold miners were purposively recruited for this study. A structured questionnaire was used to collect socio-demographic information from the participants. Work place assessment and interviews were conducted. Urine samples were analysed for protein; blood was analysed for mercury and creatinine. Estimated glomerular filtration rate (eGFR) was calculated using the chronic kidney disease-epidemiology collaboration (CKD-EPI) equation.

**Results:**

Of the 110 participants, 61(55.5%) exceeded the occupational exposure threshold (blood mercury <5μg/L). Urine protein (41.72±68.34, P<0.0001), serum creatinine (2.24±1.19, P<0.0001) and blood mercury (18.37±10.47, P<0.0001) were significantly elevated among the exposed group compared to the non-exposed group. However, the exposed group had a significantly reduced eGFR (P<0.0001). There was a significant correlation (r=0.7338, p<0.0001) between blood mercury concentration and urine protein concentration. An increase in blood mercury correlated negatively (r = −0.8233, P<0.0001) with eGFR among the exposed group. High urine protein (P< 0.0001) and high serum creatinine (P< 0.0001) were significantly associated with increased mercury exposure. Increased mercury exposure was significantly associated with burning of amalgam (P=0.0196), sucking of excess mercury (P=0.0336), longer work duration (P=0.0314) and low educational background (P=0.0473).

**Conclusion:**

Small scale miners at the Bibiani work site are exposed to excess mercury. Proteinuria and reduced eGFR is common in mine workers exposed to excess mercury. We found poor PPE compliance among the study population.

## Background

The Artisanal Small Scale Mining (ASGM) sector is very active in Ghana, and its importance is steadily growing [1]. Several health concerns exists in small-scale gold mining communities such as exposure to mercury, dust and noise, unsanitary working conditions and lack of personal protective equipment for gold mining [2].

ASGMs use mercury amalgamation to produce gold, a process which puts them at risk of exposure to mercury [2]. Exposure to mercury can occur through routes such as dermal, oral, and inhalation. Inhalation of significant concentrations of mercury vapour during processing and extraction of gold is the main route of exposure [3]. If ingested, quicksilver which is liquid at room temperature has very low toxicity because it is not absorbed by the gastrointestinal tract and is eliminated completely in the stool. Mercury is known to be toxic even at low concentrations [4]. High levels of mercury exposure deplete the amount of cellular selenium available for the biosynthesis of thioredoxin reductase and other selenoenzymes that prevent and reverse oxidative stress [5]. This can increase oxidative stress on visceral organs such as the liver and the kidney leading to potential organ damage. The kidneys are one of the main target organs for elemental mercury, with high accumulation especially in the areas of the proximal tubule [6]. Experimental studies carried out in animals revealed immunologically mediated glomerulonephritis after exposure to mercury [6] .

An estimated 1 million Ghanaians are directly involved in small-scale mining [7]. In Ghana, the main environmental problems associated with ASGM activities are mercury pollution from gold processing, ecosystems destruction and environmental degradation [8]. The small-scale gold miners in the mining site at Bibiani are known to handle the mercury without the use of appropriate PPEs like nasal and skin protectors. Occupationally, those involved in small-scale gold mining (both directly and indirectly) may inhale high levels of elemental mercury which is readily absorbed into the blood stream [2]. Prolonged exposure to mercury vapour at concentrations greater then 100 mg/m^3^ usually results in insidious onset of symptoms which when not attended to may lead to a chronic effect [9]. In Ghana, studies conducted among artisanal and small scale gold miners focused on various factors ranging from environmental and neurotoxic health risk [10], ASGM and living conditions [11] through to regulation of ASGM [12]. However, none considered the effect of mercury exposure on renal function especially in a population that works without recourse to safety procedures. Thus, we sought to assess the effect of mercury exposure on the renal function of small-scale miners in the Bibiani District of the Western region of Ghana.

## Methodology

### Study area

**Fig. 1:**
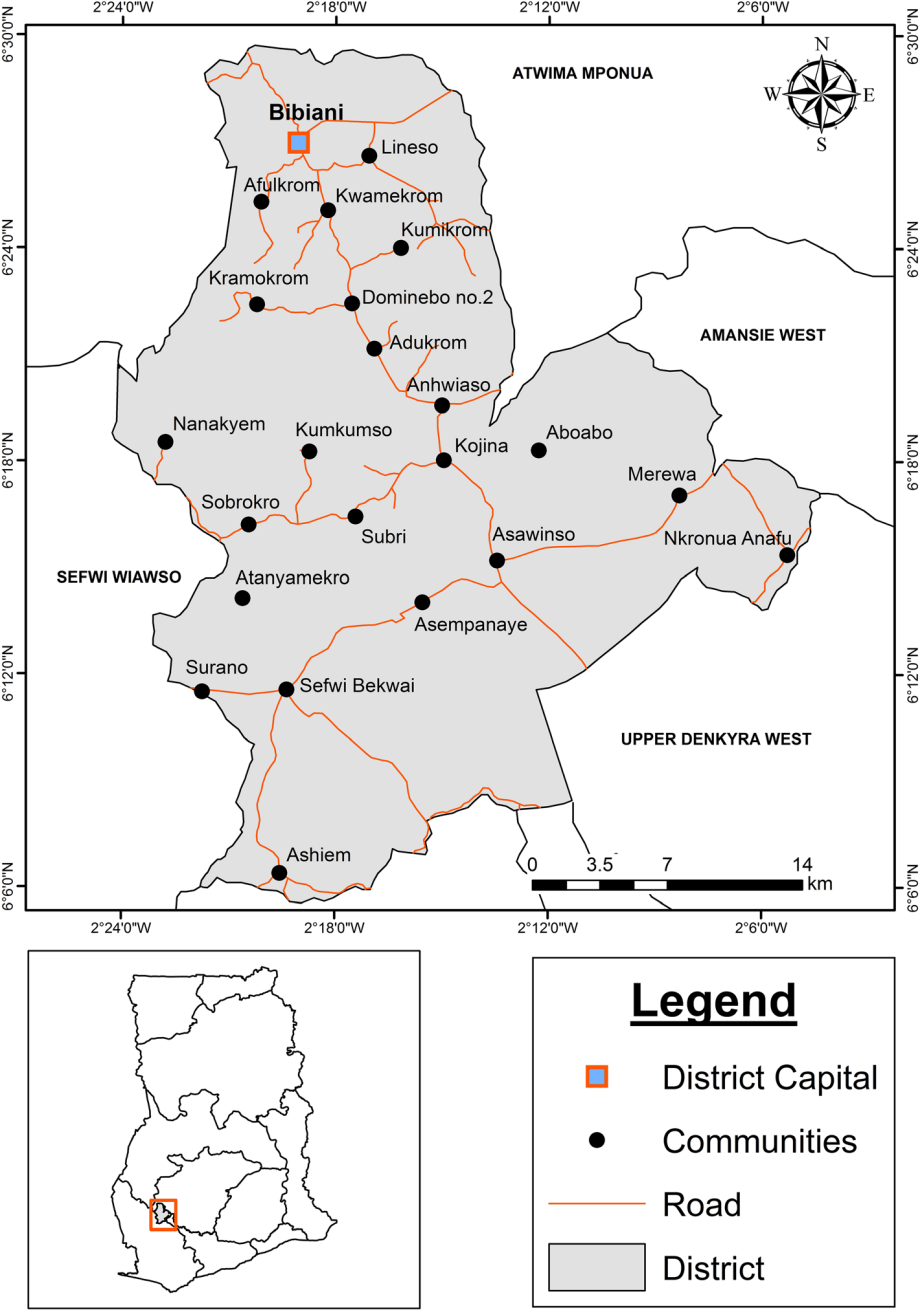
District Map of Bibiani -Anhwiaso – Berkwai, with the sampling site indicated in red

This cross-sectional study was conducted in Bibiani, an old gold mining town, in the Bibiani -Anhwiaso - Berkwai District in the North-Western part of the Western Region of Ghana (Fig. 1). Bibiani, the district capital, is 88 km from Kumasi in the Ashanti Region and 356 km from the western regional capital, Takoradi. The town has a large gold mining company called Mensin Gold Mining Company.

There are two small-scale gold mining sites in Bibiani namely Donkoto and Zongo sites respectively. The actual gold extraction is done at the Donkoto site. Zongo site is situated near a lake called Amponsem where the crushing, milling, washing, panning and amalgamation processes are done. The Zongo site is about 390 yards (380 m) in length and the width not between 150 yards (140 m) and 300 yards (290 m).

### Study population and sampling

We purposively sampled 110 male small scale gold miners staying in Bibiani. All the participants were actively involved in small-scale gold mining at the time of the study.

#### Inclusion criteria

The small-scale miners were recruited based on the following criteria: at least 1-year exposure to mercury; no history of occupational exposure to lead, cadmium and other nephrotoxic substances; no evidence of haematuria, pyuria, glycosuria or urinary tract infection in the first two years of occupational exposure to mercury; no history of renal diseases, diabetes mellitus and no evidence of consumption of analgesics and antibiotics usage two weeks prior to the examination.

### Exclusion criteria

Small-scale miners who failed to give an informed consent and persons not engaging in small scale gold mining in Bibiani were excluded from the study. Small-scale miners that had a history of occupational exposure to lead, cadmium and other nephrotoxic substances, disease conditions and infections that can interfere with urinary protein estimation and consumption of analgesics and antibiotics two weeks prior to the examination were also excluded from the study. Weight lifters at the mine site were also excluded from the study.

#### Data collection

A pre-tested structured-questionnaire was administered to the 110 consenting participants to collect their socio-demographic characteristics, occupational exposure and safety, and presence of signs and symptoms suggestive of mercury exposure.

### Urine sample collection

Twenty (20)-50 ml of random spot urine samples was collected from each participant at the work site into clean plastic bottles. All urine containers were properly labelled using the identifying numbers of participants. Safety precautions were employed in the collection of the urine samples to avoid contamination.

### Blood sample collection and transportation

Five (5) mls of blood sample was collected using sterilized syringes and needles. Three (3) mls of the blood sample was put into tripotassium ethylenediaminetetracetic acid (K_3_ EDTA) tubes and the rest into serum separator tubes. Samples were kept inside a cold plastic box containing ice packs in the field. All the samples were transported to the Laboratory at Bibiani District Hospital. Blood samples in the serum separator tubes were centrifuged at 1000 rpm for 10 min to obtain the serum which was adequately labelled and stored at -20 °C. Samples were then kept on ice packs and were transported to the Research Laboratory of the Medical Laboratory Technology Department, University of Cape Coast.

### Blood sample preparation and transportation

#### Wet digestion

The test solutions for blood mercury analysis were prepared at the Research Laboratory in the School of Agricultural of the University of Cape Coast. Two (2) grams of blood sample was transferred to the bottom of a thick-walled volumetric digestion flask, 6 ml of conc. HNO_3,_ and 1 ml of 30% H_2_O_2_ were added in turn and the mixture was heated at a temperature of 45 °C inside a block digester machine (model: EFA-5UDRVW-8) for 3 h. The mixture was allowed to cool and distilled water was added to make a fixed volume of 30 ml, it was well mixed, and the resulting solution was used as the sample test solution.

Samples were kept inside mercury free plastic containers and transported to Ghana Atomic Energy Commission (GAEC) in Accra for mercury analysis. The Cold Vapour Atomic Absorption Spectrophotometer (CVAAS), machine (varian model AA240FS), with a detection limit of 0.01μg/L was used for the analysis. The certified reference materials (CRM) used was obtained from TraceCERT®, SIGMA-ALDRICH®, USA. Individuals with mercury levels ≥5.0 μg/L were considered as occasionally exposed and those with mercury levels <5.0 μg/L were considered as non-exposed [13–15]

### Urine protein determination

Urine protein was measured immediately after collection. Proteinuria was assessed semi-quantitatively using dipstick. This was converted into a continuous scale by categorizing as negative (less than 10 mg/dL), trace (10 to 20 mg/dL), +1 (30 mg/dL), +2 (100 mg/dL), +3 (300 mg/dL) or +4 (1000 mg/dL) [16, 17].

Serum creatinine measurement/eGFR estimation

Serum creatinine was estimated with an automated chemistry analyzer (model: UV-mini-1240) with a detection limit of 0.1umol/L was used at the University of Cape Coast Hospital Laboratory for the measurement of serum creatinine levels. The principle for estimation was based on the Jaffe’s technique [18]. eGFR was estimated using the CKD-EPI eq. [19]

#### Data analysis

Data was sorted, entered and analyzed using Graph pad prism version 6.01. Exploratory analysis was carried out to obtain descriptive statistics such as frequencies, percentages, Mean ± Standard Deviation(SD), figures and tables. Kruskal Wallis test was used to compare the difference between the mercury concentrations, urine protein and estimated glomerular filtration rates among study participants based on their work durations. Spearman *rho* moment correlation analysis was done to determine correlation between mercury concentration and urine protein as well as the estimated glomerular filtration rate of the occupationally exposed participants. Mann-Whitney test was used to compare the renal function indicators of the occupationally exposed and non-exposed participants respectively. Fisher’s Exact test was used to estimate the Odds Ratio for the multivariate logistic regression of various occupational activities and educational status as well as renal biomarkers associated with mercury exposure. Multivariate logistic regression was used to estimate the age adjusted odds ratio for the renal biomarkers. Significance level was determined at *p* < 0.05.

## Results

Table 1 compares various socio-demographic parameters and biochemical characteristics of study participants stratified by exposure and non-exposure to mercury. Compared to the non-exposed group, exposed participants were married (*P* = 0.0202), had worked for a longer duration (*P* = 0.0316) and had higher levels of urine protein (*P* < 0.0001) and creatinine (*P* < 0.0001). However, eGFR, was significantly reduced (*P* < 0.0001) among the small-scale miners who were exposed.

**Table 1:** Socio-demographics and renal characteristics of the study participants

Variable	Exposed (%) *N* = 61	Non-exposed (%) *N* = 49	*p*-value
*Age (Years)*	35.77 ± 11.61	33. 96 ± 9.678	0.3838
≤19	2 (3.3)	4 (8.2)	0.2863
20–39	40 (65.6)	36 (73.5)	
40–59	17 (27.8)	7 (14.2)	
≥60	2 (3.3)	2 (4.1)	
*Marital status*			0.0202
Married	41 (67.2)	19 (38.8)	
Divorced	2 (3.3)	1 (2.0)	
Single	17 (27.8)	27 (55.1)	
Widowed	1 (1.6)	2 (4.1)	
*Highest level of education*			0.1684
Uneducated	14 (23.0)	6 (12.2)	
Primary	31 (50.8)	23 (46.9)	
Secondary	16 (26.2)	20 (40.8)	
*Ethnicity*			0.1382
Akan	45 (73.8)	32 (65.3)	
Dagomba	10 (16.3)	5 (10.2)	
Frafra	4 (6.6)	5 (10.2)	
Grussi	2 (3.3)	7 (14.3)	
*Work duration (Years)*	14.72 ± 10.67	10.77 ± 7.305	0.0316
<5	9(14.75)	11(22.44)	0.3287
≥5	52(85.25)	38(77.55)	
Work duration(hours)	9.39 ± 2.73	9.425 ± 2.27	0.942
Urine Protein(mg/dL)	41.72 ± 68.34	0.6122 ± 3.00	< 0.0001
Negative	20(32.79)	45(91.84)	<0.0001
Positive	41(67.21)	4(8.16)	
Creatinine(μmol*/L*)	2.24 ± 1.19	0.974 ± 0.184	< 0.0001
eGFR(*mL/min/1.73m* ^*2*^ *)*	57.02 ± 29.57	120.4 ± 20.57	< 0.0001
Blood mercury(μg/L)	18.37 ± 10.47	2.90 ± 1.387	< 0.0001

eGFR = Estimated glomerular filtration rate

The most common symptoms of mercury exposure among the study participants were itchy eyes [85(77.270)], fatigue [84(76.4)] and persistent headache [82(74.5)]. Hair loss was least reported among the various signs and symptoms assessed. However, there was no significance difference(*P* > 0.005) in the reported signs and symptoms when the exposed were compared to the non-exposed (Table 2).

**Table 2:** Frequency of reported signs and symptoms of mercury exposure among study participants

Variable	Exposed (%) *N* = 61(55.45)	Non-Exposed (%) *N* = 49(44.45)	Total Prevalence *N* = 110(100%)	*P*-Value
Skin Rashes			62(56.40)	0.3112
Yes	37(60.66)	25(51.02)		
No	24(39.34)	24(48.98)		
Frequent cough			70(63.6)	0.2349
Yes	42(68.85)	28(57.14)		
No	19(31.15)	21(42.85)		
Persistent Fever			73(66.4)	0.8421
Yes	41(67.21)	32(65.31)		
No	20(32.79)	17(34.69)		
Persistent Headache			82(74.5)	0.6595
Yes	47(77.05)	35(71.42)		
No	14(22.95)	13(27.66)		
Metallic Taste			60(54.5)	1.000
Yes	31(50.82)	29(59.18)		
No	30(49.18)	19(40.82)		
Fatigue			84(76.4)	1.000
Yes	47(77.05)	37(75.51)		
No	14(22.95)	12(24.49)		
Muscle ache			60(54.5)	0.3381
Yes	36(59.02)	24(48.98)		
NO	25(40.98)	25(51.02)		
Numbness			45(40.9)	0.1240
Yes	29(47.54)	16(32.65)		
No	32(52.46)	33(67.35)		
Hair Loss			5(4.50)	1.000
Yes	3(4.92)	2(4.08))		
No	58(95.08)	47(95.92)		
Itchy Eyes			85(77.27)	0.8195
Yes	48(78.69	37(75.51)		
No	13(21.31)	12(24.49)		

The total (percent) absolute non-compliance of appropriate use of the protective clothing or equipment (participant who never used any of the protective clothing or equipment at all) was 86.55%. The use of rubber aprons [109(99.09)] and leather boots [109(99.09)] were the least complied protection measures, whilst the use of mercury containers 47(42.73) was the most complied protection measure (Table 3).

**Table 3:** Frequency of use of various protective clothing among the study participants

	Mercury exposure			
Variable	Exposed (%) ≥5μg/L	Non-Exposed (%) <5μg/L	Total frequency (%)	*P*-Value
*Rubber aprons*			109(99.09)	1.000
Don’t use	60(98.36)	49(100)		
Seldom use	1(1.64)	0(0)		
*Mercury Containers*			47(42.73)	0.2095
Don’t use	29(47.54)	18(36.73)		
Always use	28(45.90)	23(46.94)		
Seldom use	4(6.55)	8(16.34)		
*Nose mask*			103(93.64)	0.3270
Don’t use	59(96.72)	44(89.80)		
Always use	1(1.64)	2(4.08)		
Seldom use	1(1.64)	3(6.12)		
*Rubber gloves*			108(98.18)	0.5014
Don’t use	59(96.72)	49(100)		
Seldom use	2(3,28)	0(0)		
*Leather boots*			109(99.09)	1.000
Always use	1(1.64)	0(0)		
Don’t use	60(98.36)	49(100)		

Multivariate logistic regression analysis of various occupation activities associated with increased mercury exposure showed that burning of amalgam [OR = 4.212(95%CI = 1.238–8.337), *P* = 0.0196], sucking of excess mercury [OR = 3.352 (95%CI = 0.6210–18.10), *P* = 0.0336)], longer work duration [OR = 0.379 (0.158–0.91), *P* = 0.0314] and low educational status were significantly associated with increased mercury exposure. (Table 4)

**Table 4:** Multivariate logistic regression analysis of various occupational activities and educational status associated with mercury exposure

Variable	Exposed *N* = 61	Non-exposed *N* = 49	Crude OR(95%CI)	*P* value
*Gold Amalgamation*				1.0000
Yes	59 (96.7)	48 (97.9)	0.6146 (0.054–6.989)	
NO	2 (3.3)	1 (2.1)	*	
*Burning of Amalgam*				0.0196
Yes	53 (86.9)	33 (67.3)	3.212 (1.238–8.337)	
No	8 (13.1)	16 (32.7)	*	
*Smelting of Gold*				1.000
Yes	58 (86.9)	47 (95.9)	0.8227 (0.1319–5.13)	
NO	3 (13.1)	2 (4.1)	*	
*Transportation of mercury*				0.2384
Yes	59 (96.7)	44 (89.8)	3.352 (0.6210–18.10)	
NO	2 (3.3)	5 (10.2)	*	
*Standing in pool of water stream*				0.1064
Yes	58 (86.9)	42 (85.7)	3.222 (0.7867–13.20)	
No	3 (13.1)	7 (14.3)	*	
*Sucking of excess mercury*				0.0336
Yes	53 (86.9)	34 (69.4)	2.923 (1.119–7.636)	
No	8(3.3)	15 (30.6)	*	
*Work Duration(years)*				0.0314
<5	11 (18.0)	18 (36.7)	*	
≥5	50 (82.0)	31 (63.3)	2.639(1.101–6.32)	
*Fish consumption from lake*				1.0000
Yes	33 (54.1)	27 (55.1)	0.960 (0.451–2.04)	
NO	28 (45.9)	22 (44.9)	*	
*Previous work*				0.8382
Yes	19 (31.1)	17 (34.7)	0.8515 (0.38–1.89)	
No	42 (68.9)	32 (65.3)	*	
*Place of mercury storage*				
Don’t store Mercury	3 (4.9)	2 (4.1)	*	
Home	43 (70.5)	31 (63.3)	0.925 (0.146–5.87)	1.000
Mine Site	15 (24.6)	16 (32.6)	0.625 (0.091–4.27)	1.000
*Knowledge of mercury intoxication*				0.8447
Yes	24 (39.3)	18 (36.7)	1.117 (0.514–2.42)	
NO	37 (60.7)	31 (63.3)	*	
*Highest level of education*				
Uneducated	14 (23.0)	5 (10.2)	3.675 (1.09–12.34)	0.0473
Primary	31 (50.8)	23 (47.0)	1.77 (0.7598–4.12)	0.206
Secondary	16 (26.2)	21 (42.8)	*	

Age-AOR = Age Adjusted Odds Ratio

High urine protein [OR = 50.29(95%CI = 10.97–230.53, *P* < 0.0001], and elevated serum creatinine [OR = 101.6(95%CI = 25.45–405.7), *P* < 0.0001) are associated with high mercury exposure. Reduced eGFR [OR = 263.2(95%CI = 48.79–1420), *P* < 0.0001] was associated with increased mercury exposure among small scale miners when adjusted for age (Table 5).

**Table 5:** Multivariate logistic regression analysis of renal biomarkers associated with mercury exposure

	Mercury Exposure			
Variable	Exposed (%)	Non -exposed (%)	Age-AOR (95% CI)	*P*-value
*Urine protein (mg/dl)*				
Normal (<10)	20 (32.79)	47 (95.9)	*	
High (≥10)	41 (67.21)	2 (4.1)	50.29 (10.97–230.53)	< 0.0001
*eGFR (mL/min/1.73m* ^*2*^ *)*				
Normal (>90)	5 (8.2)	47 (95.91)	*	
Low (≤90)	56(91.8)	2 (4.1)	263.2(48.79–1420)	<0.0001
*Creatinine (*μmol*/L)*				
Normal (53–106)	8 (13.1)	46 (75.4)	*	
High (>106)	53 (86.9)	3 (24.6	101.06 (25.23–404.85)	< 0.0001

## Discussion

We sought to assess the effect of mercury exposure on the renal biomarkers and level of PPE compliance of ASGMs working at the Bibiani mine site. Our findings showed that increased proteinuria and reduced eGFR were common among the miners exposed to higher levels of mercury; increased renal abnormalities were related to longer working duration and low PPE compliance.

Majority of the participants were exposed to mercury above the exposure threshold in consonance with earlier studies conducted in South Africa, Brazil and Ghana which reported 50%, 48.3% and 46.65 of the participants having elevated urine mercury concentration respectively [14, 20, 21]. Urine mercury levels have been shown to directly correlate strongly with blood mercury [22] and thus urine concentration usually reflects blood mercury concentration. De Kom et al. also reported high serum mercury levels in a case-control study among Maroon workers in small-scale gold mines in Suriname [23].

**Fig. 2:**
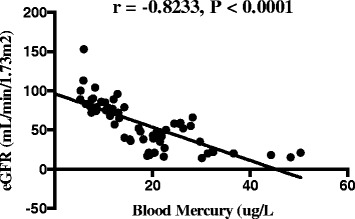
Scatter plot and Spearman rho moment correlation between blood mercury and eGFR of exposed participantsBlood mercury levels correlated positively (*r* = 0.7338, *p* < 0.0001) with proteinuria but negatively (*r* = −0.8233, *p* < 0.0001) with eGFR (Figs. 2 and 3)

The kidneys are the main target organs for elemental mercury besides the central nervous system [24]. The reduced glomerular filtration rate among the study participants, as well as the elevated serum creatinine is an indication of renal function impairment. Augusti et al., (2007) [25] on the assessment of the effects of astaxanthin on kidney function impairment and oxidative stress induced by mercury chloride in rats also reported a reduced glomerular filtration as a consequence of elevated serum creatinine after mercury injection. In line with the findings of Kobal and co [26] we reported a negative correlation between blood mercury levels and eGFR (Fig. 2) among miners intermittently exposed to mercury.

**Fig. 3:**
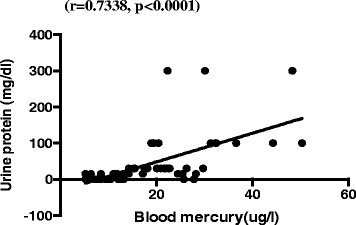
Scatter plot and Spearman rho moment correlation between blood mercury and urine protein of exposed participants

Proteinuria is a clinical manifestation of mercury intoxication due to elemental inorganic and ethyl mercury [27]. In agreement with earlier studies in Sekotong, West Lambork-Indonesia [16, 28], and in Irija mines in Nigeria [16] we noted a significant increase in proteinuria among mine workers exposed to higher levels of mercury (Fig. 3). Persistent proteinuria indicates kidney disease [29] and within the kidney, the pars recta of the proximal tubule has been known to be the most vulnerable segment of the nephron that is susceptible to the toxic effects of mercury [30].

The extent of renal dysfunction has been severally related to extent of mercury exposure, route of exposure, the state of the mercury and the duration of exposure [31]. Franko and Dodic-Fikfak [28] reported the duration for the commencement of clinical manifestation among small scale gold miners to be 15 years in a case control-study in Idrija mercury mines. Similarly, we report a mean duration of 14.72 years as the duration for the presentation of renal abnormalities with participants who have worked for more than 5 years at the Bibiani mines being likely to present with elevated blood mercury above the occupational threshold.

Various signs and symptoms ranging from skin rashes through persistent headaches to hair loss have been reported as associated with the use of mercury in small-scale mining. These may vary from location to location as well as extent of exposure [2]. Whiles we report “itchy eyes” and fatigue as the most common sign and symptom suggestive of mercury exposure, Mensah in 2012, reported complaints of skin rashes, red eyes and metallic taste to be associated with mercury exposure among small scale miners in Prestea, Ghana [2] . Other studies have reported red eyes and conjunctivitis among individuals exposed to high concentration of elemental mercury vapours [32]. This could be attributed to continuous higher occupational exposure to elemental mercury vapour.

According to WHO, the main route of mercury exposure in ASGM is inhalation of vaporized elemental mercury released during amalgam smelting [33]. However, we observed a very low compliance with PPE use among our participants (Table3). In line with a study by Hilson et al., [34] in the Talensi-Nabdam District of Ghana, we recorded limited use of PPE among small- scale miners even though some were aware of the of the health risks associated with mercury thus corroborating the findings of Rojas et al., [35]. The high non-compliance with the use of PPEs may be attributed to the low level of education and apathy, since majority of the participants were either uneducated or had only basic education. Also, most of the participants did not have any form of occupational safety training in mercury processing and handling. The clinical interpretations of our findings may be limited due to the lack of pure controls who are not miners and our inability to measure urine mercury which is a better biomarker for chronic mercury exposure. A study considering the specific dose of mercury exposure will provide further information on the duration and extent of renal derangement.

## Conclusion

We conclude that small scale miners at Bibiani work site are occupationally exposed to excess mercury which may affect their renal integrity. PPE compliance is low among the study participants.

## Abbreviations

ASGM: Artisanal Small Scale Mining
eGFR: Estimated Glomerular Filtration Rate
PPE: Personal Protective Equipment
